# 2-Hy­droxy-2-trifluoro­methyl-3,4-dihydro-2*H*-1-benzopyran-4-one

**DOI:** 10.1107/S1600536812029170

**Published:** 2012-06-30

**Authors:** Abdullah M. Asiri, Hassan M. Faidallah, Khalid A. Alamry, Seik Weng Ng, Edward R. T. Tiekink

**Affiliations:** a Center of Excellence for Advanced Materials Research (CEAMR), King Abdulaziz University, PO Box 80203, Jeddah 21589, Saudi Arabia; b Chemistry Department, Faculty of Science, King Abdulaziz University, PO Box 80203, Jeddah 21589, Saudi Arabia; c Department of Chemistry, University of Malaya, 50603 Kuala Lumpur, Malaysia

## Abstract

The heterocyclic ring in the title compound, C_10_H_7_F_3_O_3_, has a half-boat conformation with the hy­droxy-bearing C atom lying 0.595 (3) Å out of the plane of the five remaining atoms (r.m.s. deviation = 0.022 Å) in the direction of the hy­droxy O atom. Linear supra­molecular chains along the *a* axis, sustained by O—H⋯O hydrogen bonds between the hy­droxy H and ketone O atoms, feature in the crystal packing. These chains are connected into a three-dimensional architecture by C—H⋯O and C—H⋯F contacts.

## Related literature

For an example of an anti­cipated product formed between the reaction of bis­(ethyl­idene)ethane-1,2-diamine with an anhydride, see: Asiri *et al.* (2011 ▶). For the crystal structure of a related compound, see: Wang *et al.* (1999 ▶).
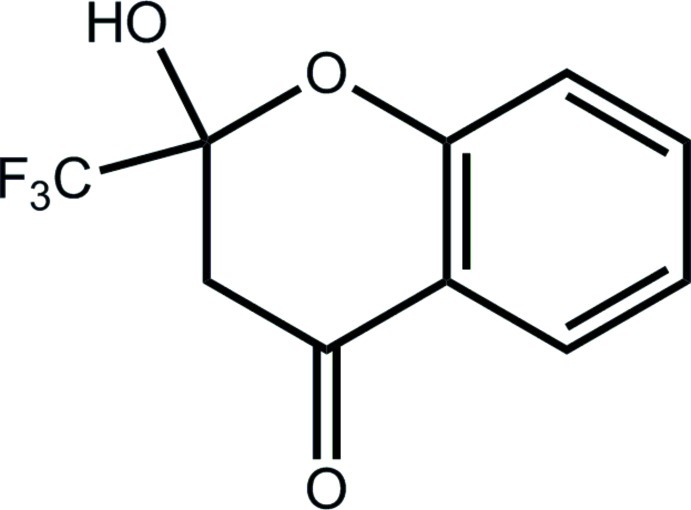


## Experimental

### 

#### Crystal data

C_10_H_7_F_3_O_3_
*M* *_r_* = 232.16
Triclinic, 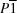

*a* = 5.9516 (5) Å
*b* = 8.5188 (7) Å
*c* = 10.2036 (8) Å
α = 66.985 (8)°
β = 80.380 (7)°
γ = 78.311 (7)°
*V* = 464.05 (7) Å^3^
*Z* = 2
Mo *K*α radiation
μ = 0.16 mm^−1^
*T* = 100 K
0.30 × 0.30 × 0.15 mm

#### Data collection

Agilent SuperNova Dual diffractometer with an Atlas detector
Absorption correction: multi-scan (*CrysAlis PRO*; Agilent, 2012 ▶) *T*_min_ = 0.528, *T*_max_ = 1.000
3161 measured reflections
2126 independent reflections
1665 reflections with *I* > 2σ(*I*)
*R*_int_ = 0.027

#### Refinement

*R*[*F* ^2^ > 2σ(*F* ^2^)] = 0.051
*wR*(*F* ^2^) = 0.171
*S* = 1.06
2126 reflections
149 parameters
H atoms treated by a mixture of independent and constrained refinement
Δρ_max_ = 0.58 e Å^−3^
Δρ_min_ = −0.30 e Å^−3^

### 

Data collection: *CrysAlis PRO* (Agilent, 2012 ▶); cell refinement: *CrysAlis PRO*; data reduction: *CrysAlis PRO*; program(s) used to solve structure: *SHELXS97* (Sheldrick, 2008 ▶); program(s) used to refine structure: *SHELXL97* (Sheldrick, 2008 ▶); molecular graphics: *ORTEP-3 for Windows* (Farrugia, 1997 ▶) and *DIAMOND* (Brandenburg, 2006 ▶); software used to prepare material for publication: *publCIF* (Westrip, 2010 ▶).

## Supplementary Material

Crystal structure: contains datablock(s) global, I. DOI: 10.1107/S1600536812029170/su2468sup1.cif

Structure factors: contains datablock(s) I. DOI: 10.1107/S1600536812029170/su2468Isup2.hkl

Supplementary material file. DOI: 10.1107/S1600536812029170/su2468Isup3.cml

Additional supplementary materials:  crystallographic information; 3D view; checkCIF report

## Figures and Tables

**Table 1:** Hydrogen-bond geometry (Å, °)

*D*—H⋯*A*	*D*—H	H⋯*A*	*D*⋯*A*	*D*—H⋯*A*
O3—H3*o*⋯O2^i^	0.86 (3)	1.97 (3)	2.768 (2)	154 (3)
C2—H2⋯O1^ii^	0.95	2.60	3.444 (3)	148
C3—H3⋯F3^iii^	0.95	2.52	3.338 (3)	144
C8—H8*A*⋯F1^iv^	0.99	2.51	3.033 (2)	113
C8—H8*B*⋯O3^v^	0.99	2.56	3.547 (2)	175

Symmetry codes: (i) 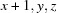; (ii) 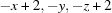; (iii) 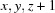; (iv) 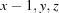; (v) 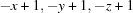.

**Fig. 1.**
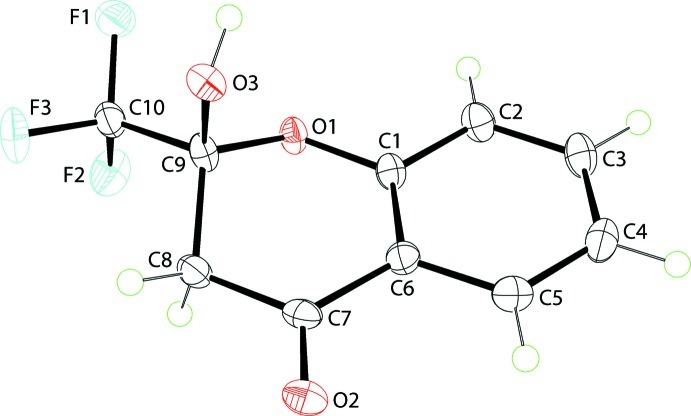
The molecular structure of (I) showing the atom-labelling scheme and displacement ellipsoids at the 50% probability level.

**Fig. 2.**
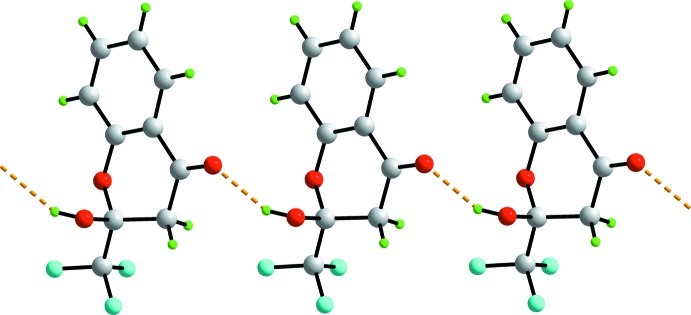
A view of the linear supramolecular chain along the a axis in (I) mediated by O—H···O hydrogen bonds shown as orange dashed lines.

**Fig. 3.**
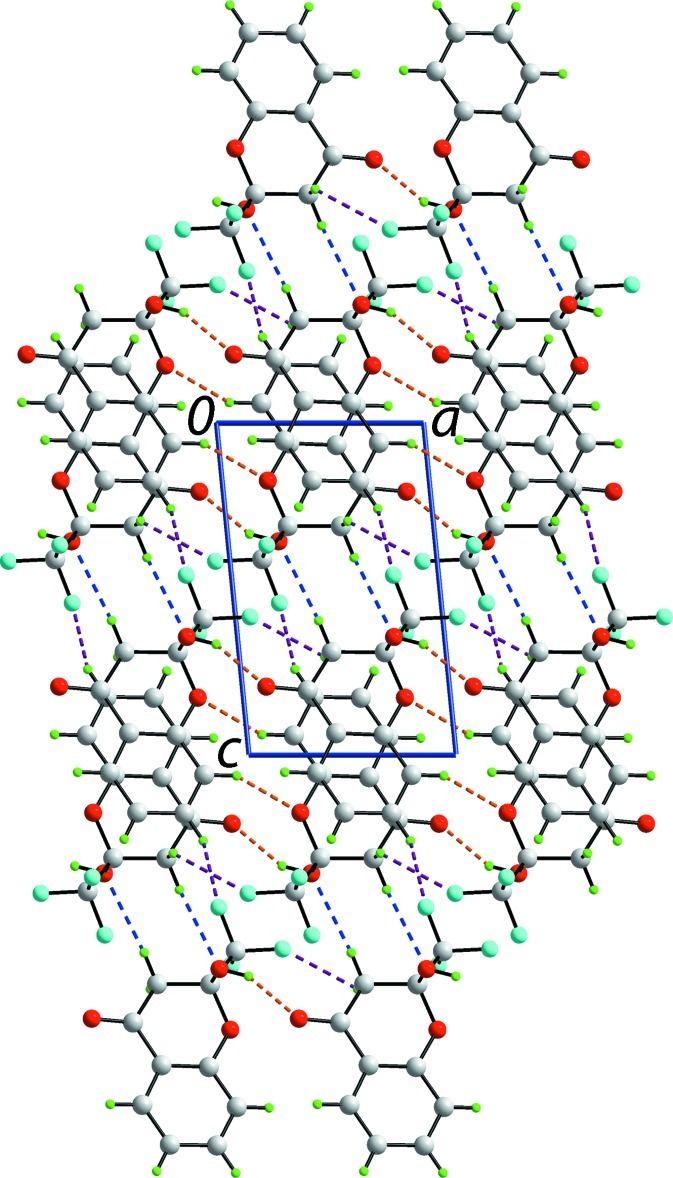
A view in projection along the b axis of the unit-cell contents of (I). The O—H···O, C—H···O and C—H···F interactions are shown as orange, blue and purple dashed lines, respectively.

## supplementary crystallographic information

### Comment

The title compound, (I), was isolated unexpectedly from a reaction between *N*,*N*'-bis[1-(*p*-hydroxyhenyl)ethylidene]ethane-1,2-diamine and trifluoroacetic anhydride to yield the anticipated di-substituted ethylenediamine derivative in accord with literature precedents (Asiri *et al.*, 2011).

In (I), Fig. 1, the heterocyclic ring has a half-boat conformation with the hydroxy bearing C9 atom lying 0.595 (3) Å out of the plane of the five remaining atoms [r.m.s. deviation = 0.022 Å] in the direction of the hydroxy O3 atom. A similar conformation was found in a literature precedent with phenyl rather than CF_3_ and with OH and two OMe substituents on the benzene ring (Wang *et al.*, 1999).

In the crystal, linear supramolecular chains sustained by O—H···O hydrogen bonds between the hydroxy H and ketone-O atoms (Table 1), are formed along the *a* axis (Fig. 2). These are connected into a three-dimensional architecture by C—H···O and C—H···F contacts (Fig. 3 and Table 1).

### Experimental

A mixture of *N*,*N*'-bis[1-(*p*-hydroxyhenyl)ethylidene]ethane-1,2-diamine (0.01 *M*) in THF (30 ml) and trifluoroacetic anhydride (0.025 *M*) was refluxed for 2 h. The solid which separated on cooling was recrystallized from its ethanol solution. *M*. pt: 477–478 K. Yield: 70%.

### Refinement

Carbon-bound H-atoms were placed in calculated positions [C—H = 0.95–0.99 Å, *U*_iso_(H) = 1.2*U*_eq_(C)] and were included in the refinement in the riding model approximation. The oxygen-bound H-atom was located in a difference Fourier map and was refined freely.

### Crystal data

**Table:**

C_10_H_7_F_3_O_3_	*Z* = 2
*M**_r_* = 232.16	*F*(000) = 236
Triclinic, *P*1	*D*_x_ = 1.661 Mg m^−^^3^
Hall symbol: -P 1	Mo *K*α radiation, λ = 0.71073 Å
*a* = 5.9516 (5) Å	Cell parameters from 1358 reflections
*b* = 8.5188 (7) Å	θ = 2.6–27.5°
*c* = 10.2036 (8) Å	µ = 0.16 mm^−^^1^
α = 66.985 (8)°	*T* = 100 K
β = 80.380 (7)°	Block, colourless
γ = 78.311 (7)°	0.30 × 0.30 × 0.15 mm
*V* = 464.05 (7) Å^3^	

### Data collection

**Table:**

Agilent SuperNova Dual diffractometer with an Atlas detector	2126 independent reflections
Radiation source: SuperNova (Mo) X-ray Source	1665 reflections with *I* > 2σ(*I*)
Mirror monochromator	*R*_int_ = 0.027
Detector resolution: 10.4041 pixels mm^-1^	θ_max_ = 27.6°, θ_min_ = 2.6°
ω scan	*h* = −7→7
Absorption correction: multi-scan (*CrysAlis PRO*; Agilent, 2012)	*k* = −8→11
*T*_min_ = 0.528, *T*_max_ = 1.000	*l* = −12→13
3161 measured reflections	

### Refinement

**Table:**

Refinement on *F*^2^	Primary atom site location: structure-invariant direct methods
Least-squares matrix: full	Secondary atom site location: difference Fourier map
*R*[*F*^2^ > 2σ(*F*^2^)] = 0.051	Hydrogen site location: inferred from neighbouring sites
*wR*(*F*^2^) = 0.171	H atoms treated by a mixture of independent and constrained refinement
*S* = 1.06	*w* = 1/[σ^2^(*F*_o_^2^) + (0.1*P*)^2^] where *P* = (*F*_o_^2^ + 2*F*_c_^2^)/3
2126 reflections	(Δ/σ)_max_ = 0.001
149 parameters	Δρ_max_ = 0.58 e Å^−^^3^
0 restraints	Δρ_min_ = −0.30 e Å^−^^3^

### Special details

**Table:**

Geometry. All e.s.d.'s (except the e.s.d. in the dihedral angle between two l.s. planes) are estimated using the full covariance matrix. The cell e.s.d.'s are taken into account individually in the estimation of e.s.d.'s in distances, angles and torsion angles; correlations between e.s.d.'s in cell parameters are only used when they are defined by crystal symmetry. An approximate (isotropic) treatment of cell e.s.d.'s is used for estimating e.s.d.'s involving l.s. planes.
Refinement. Refinement of *F*^2^ against ALL reflections. The weighted *R*-factor *wR* and goodness of fit *S* are based on *F*^2^, conventional *R*-factors *R* are based on *F*, with *F* set to zero for negative *F*^2^. The threshold expression of *F*^2^ > σ(*F*^2^) is used only for calculating *R*-factors(gt) *etc*. and is not relevant to the choice of reflections for refinement. *R*-factors based on *F*^2^ are statistically about twice as large as those based on *F*, and *R*- factors based on ALL data will be even larger.

### Fractional atomic coordinates and isotropic or equivalent isotropic displacement parameters (Å^2^)

**Table:**

	*x*	*y*	*z*	*U*_iso_*/*U*_eq_	
F1	1.0703 (2)	0.18377 (19)	0.58363 (13)	0.0304 (4)	
F2	0.8148 (2)	0.01895 (17)	0.63198 (13)	0.0313 (4)	
F3	0.7818 (2)	0.26448 (18)	0.45820 (12)	0.0319 (4)	
O1	0.7796 (2)	0.16833 (18)	0.82675 (13)	0.0178 (3)	
O2	0.1156 (2)	0.4181 (2)	0.79358 (15)	0.0235 (4)	
O3	0.7560 (3)	0.43915 (19)	0.64399 (14)	0.0209 (4)	
C1	0.6584 (3)	0.2102 (3)	0.93909 (19)	0.0175 (4)	
C2	0.7742 (4)	0.1650 (3)	1.0596 (2)	0.0211 (5)	
H2	0.9293	0.1085	1.0621	0.025*	
C3	0.6602 (4)	0.2034 (3)	1.1755 (2)	0.0232 (5)	
H3	0.7378	0.1719	1.2583	0.028*	
C4	0.4331 (4)	0.2877 (3)	1.1729 (2)	0.0237 (5)	
H4	0.3574	0.3141	1.2531	0.028*	
C5	0.3191 (3)	0.3325 (3)	1.0531 (2)	0.0213 (5)	
H5	0.1641	0.3892	1.0514	0.026*	
C6	0.4307 (3)	0.2950 (3)	0.93380 (19)	0.0175 (4)	
C7	0.3132 (3)	0.3418 (3)	0.8052 (2)	0.0188 (4)	
C8	0.4479 (3)	0.2822 (3)	0.6894 (2)	0.0195 (4)	
H8A	0.4129	0.1672	0.7043	0.023*	
H8B	0.3997	0.3636	0.5950	0.023*	
C9	0.7043 (3)	0.2716 (3)	0.68994 (19)	0.0186 (4)	
C10	0.8435 (3)	0.1831 (3)	0.59025 (19)	0.0202 (5)	
H3o	0.893 (6)	0.430 (5)	0.666 (3)	0.064 (10)*	

### Atomic displacement parameters (Å^2^)

**Table:**

	*U*^11^	*U*^22^	*U*^33^	*U*^12^	*U*^13^	*U*^23^
F1	0.0218 (7)	0.0439 (9)	0.0346 (7)	−0.0066 (6)	0.0015 (5)	−0.0251 (7)
F2	0.0428 (9)	0.0221 (7)	0.0318 (7)	−0.0061 (6)	0.0050 (6)	−0.0156 (6)
F3	0.0443 (9)	0.0362 (8)	0.0169 (6)	0.0016 (6)	−0.0076 (5)	−0.0135 (6)
O1	0.0193 (7)	0.0196 (8)	0.0144 (6)	0.0026 (6)	−0.0048 (5)	−0.0075 (6)
O2	0.0167 (7)	0.0255 (8)	0.0282 (8)	−0.0012 (6)	−0.0067 (6)	−0.0091 (7)
O3	0.0206 (8)	0.0202 (8)	0.0234 (7)	−0.0026 (6)	−0.0062 (6)	−0.0083 (6)
C1	0.0222 (10)	0.0153 (10)	0.0148 (9)	−0.0017 (8)	−0.0037 (8)	−0.0054 (8)
C2	0.0236 (11)	0.0194 (11)	0.0204 (9)	0.0016 (8)	−0.0070 (8)	−0.0079 (8)
C3	0.0335 (12)	0.0200 (11)	0.0159 (9)	−0.0024 (9)	−0.0064 (8)	−0.0057 (8)
C4	0.0314 (12)	0.0222 (11)	0.0180 (9)	−0.0064 (9)	0.0035 (8)	−0.0094 (9)
C5	0.0189 (10)	0.0193 (11)	0.0256 (10)	−0.0029 (8)	−0.0018 (8)	−0.0085 (9)
C6	0.0182 (10)	0.0172 (10)	0.0169 (9)	−0.0043 (8)	−0.0008 (7)	−0.0057 (8)
C7	0.0161 (10)	0.0187 (10)	0.0221 (9)	−0.0067 (8)	−0.0033 (8)	−0.0055 (8)
C8	0.0181 (10)	0.0224 (11)	0.0195 (9)	−0.0040 (8)	−0.0055 (8)	−0.0073 (8)
C9	0.0218 (10)	0.0198 (10)	0.0156 (9)	0.0005 (8)	−0.0067 (8)	−0.0080 (8)
C10	0.0234 (11)	0.0224 (11)	0.0177 (9)	−0.0044 (8)	−0.0037 (8)	−0.0092 (8)

### Geometric parameters (Å, º)

**Table:**

F1—C10	1.341 (2)	C3—C4	1.395 (3)
F2—C10	1.332 (2)	C3—H3	0.9500
F3—C10	1.328 (2)	C4—C5	1.381 (3)
O1—C1	1.379 (2)	C4—H4	0.9500
O1—C9	1.419 (2)	C5—C6	1.405 (3)
O2—C7	1.223 (3)	C5—H5	0.9500
O3—C9	1.400 (2)	C6—C7	1.467 (3)
O3—H3o	0.86 (3)	C7—C8	1.512 (3)
C1—C2	1.393 (2)	C8—C9	1.511 (3)
C1—C6	1.400 (3)	C8—H8A	0.9900
C2—C3	1.382 (3)	C8—H8B	0.9900
C2—H2	0.9500	C9—C10	1.534 (3)
			
C1—O1—C9	115.51 (14)	O2—C7—C8	121.50 (17)
C9—O3—H3o	107 (2)	C6—C7—C8	115.72 (17)
O1—C1—C2	116.74 (17)	C9—C8—C7	111.33 (15)
O1—C1—C6	122.38 (16)	C9—C8—H8A	109.4
C2—C1—C6	120.89 (17)	C7—C8—H8A	109.4
C3—C2—C1	119.07 (19)	C9—C8—H8B	109.4
C3—C2—H2	120.5	C7—C8—H8B	109.4
C1—C2—H2	120.5	H8A—C8—H8B	108.0
C2—C3—C4	121.14 (18)	O3—C9—O1	111.26 (14)
C2—C3—H3	119.4	O3—C9—C8	108.60 (17)
C4—C3—H3	119.4	O1—C9—C8	111.81 (16)
C5—C4—C3	119.62 (18)	O3—C9—C10	108.97 (16)
C5—C4—H4	120.2	O1—C9—C10	104.54 (16)
C3—C4—H4	120.2	C8—C9—C10	111.61 (15)
C4—C5—C6	120.47 (19)	F3—C10—F2	107.65 (15)
C4—C5—H5	119.8	F3—C10—F1	107.39 (16)
C6—C5—H5	119.8	F2—C10—F1	106.72 (17)
C1—C6—C5	118.81 (17)	F3—C10—C9	110.52 (17)
C1—C6—C7	119.75 (17)	F2—C10—C9	112.73 (16)
C5—C6—C7	121.44 (18)	F1—C10—C9	111.56 (15)
O2—C7—C6	122.72 (18)		
			
C9—O1—C1—C2	−154.98 (17)	O2—C7—C8—C9	152.82 (19)
C9—O1—C1—C6	24.6 (2)	C6—C7—C8—C9	−30.0 (2)
O1—C1—C2—C3	−179.77 (17)	C1—O1—C9—O3	70.0 (2)
C6—C1—C2—C3	0.7 (3)	C1—O1—C9—C8	−51.6 (2)
C1—C2—C3—C4	−0.6 (3)	C1—O1—C9—C10	−172.49 (15)
C2—C3—C4—C5	0.5 (3)	C7—C8—C9—O3	−69.3 (2)
C3—C4—C5—C6	−0.5 (3)	C7—C8—C9—O1	53.8 (2)
O1—C1—C6—C5	179.84 (16)	C7—C8—C9—C10	170.52 (16)
C2—C1—C6—C5	−0.6 (3)	O3—C9—C10—F3	−63.4 (2)
O1—C1—C6—C7	0.2 (3)	O1—C9—C10—F3	177.59 (14)
C2—C1—C6—C7	179.72 (17)	C8—C9—C10—F3	56.6 (2)
C4—C5—C6—C1	0.5 (3)	O3—C9—C10—F2	176.10 (15)
C4—C5—C6—C7	−179.83 (18)	O1—C9—C10—F2	57.1 (2)
C1—C6—C7—O2	−178.94 (18)	C8—C9—C10—F2	−64.0 (2)
C5—C6—C7—O2	1.4 (3)	O3—C9—C10—F1	56.0 (2)
C1—C6—C7—C8	3.9 (3)	O1—C9—C10—F1	−63.0 (2)
C5—C6—C7—C8	−175.77 (17)	C8—C9—C10—F1	175.96 (15)

### Hydrogen-bond geometry (Å, º)

**Table:**

*D*—H···*A*	*D*—H	H···*A*	*D*···*A*	*D*—H···*A*
O3—H3*o*···O2^i^	0.86 (3)	1.97 (3)	2.768 (2)	154 (3)
C2—H2···O1^ii^	0.95	2.60	3.444 (3)	148
C3—H3···F3^iii^	0.95	2.52	3.338 (3)	144
C8—H8*A*···F1^iv^	0.99	2.51	3.033 (2)	113
C8—H8*B*···O3^v^	0.99	2.56	3.547 (2)	175

Symmetry codes: (i) *x*+1, *y*, *z*; (ii) −*x*+2, −*y*, −*z*+2; (iii) *x*, *y*, *z*+1; (iv) *x*−1, *y*, *z*; (v) −*x*+1, −*y*+1, −*z*+1.
